# Buffering Capacity in Sepsis: A Prospective Cohort Study in Critically Ill Patients

**DOI:** 10.3390/jcm8111759

**Published:** 2019-10-23

**Authors:** Ioannis Vasileiadis, Maria Kompoti, Nikoletta Rovina, Elli-Sophia Tripodaki, Christos Filis, Emmanouil Alevrakis, Anna Kyriakoudi, Magdalini Kyriakopoulou, Nikolaos Koulouris, Antonia Koutsoukou

**Affiliations:** 1 Intensive Care Unit, First Department of Respiratory Medicine, Medical School, National and Kapodistrian University of Athens, 11527 Athens, Greece; nikrovina@med.uoa.gr (N.R.); annkyr@gmail.com (A.K.); mgkyriak@gmail.com (M.K.); koulnik@med.uoa.gr (N.K.); koutsoukou@yahoo.gr (A.K.); 2 Intensive Care Unit, Thriassio General Hospital of Eleusis, 19600 Eleusis, Greece; mariakompoti@gmail.com; 3 First Department of Internal Medicine, Agios Savvas Regional Cancer Hospital, 11522 Athens, Greece; ellisophia@hotmail.com; 4 Third Department of Internal Medicine, Medical School, National and Kapodistrian University of Athens, 11527 Athens, Greece; christos.filis@gmail.com; 5 4th Department of Respiratory Medicine, Sotiria Hospital, 11527 Athens, Greece; m.alevrakis@gmail.com

**Keywords:** sepsis, buffering capacity, acid-base balance, homeostasis, central venous-arterial PCO_2_ difference, strong ion difference

## Abstract

Background: The concept of buffering generally refers to the ability of a system/organism to withstand attempted changes. For acid-base balance in particular, it is the body’s ability to limit pH aberrations when factors that potentially affect it change. Buffering is vital for maintaining homeostasis of an organism. The present study was undertaken in order to investigate the probable buffering capacity changes in septic patients. Materials and methods: This prospective cohort study included 113 ICU patients (96 septic and 17 critically-ill non-septic/controls). The buffering capacity indices were assessed upon ICU admission and reassessed only in septic patients, either at improvement or upon severe deterioration. Applying Stewart’s approach, the buffering capacity was assessed with indices calculated from the observed central venous-arterial gradients: a) ΔPCO_2_/Δ[H^+^] or ΔpH, b) ΔSID/Δ[H^+^] or ΔpH. Results: In a generalized estimating equation linear regression model, septic patients displayed significant differences in ΔPCO_2_/ΔpH [beta coefficient = –47.63, 95% CI (–80.09) – (–15.17), *p* = 0.004], compared to non-septic patients on admission. Lower absolute value of ΔPCO_2_/ΔpH (%) on admission was associated with a significant reduction in ICU mortality (HR 0.98, 95% CI: 0.97–0.99, *p* = 0.02). At septic-group reassessment (remission or deterioration), one-unit increase of ΔPCO_2_/Δ[H^+^] reduced the ICU death hazard by 44% (HR 0.56, 95% CI: 0.33–0.96, *p* = 0.03). Conclusions: In the particular cohort of patients studied, a difference in the buffering capacity was recorded between septic and non-septic patients on admission. Moreover, buffering capacity was an independent predictor of fatal ICU outcome at both assessments, ICU-admission and sepsis remission or deterioration.

## 1. Introduction

In all body tissues, the microcirculation represents an exchange site for nutrients, oxygen and waste products; being crucial for the preservation of structural and functional integrity of all organs and systems, it ultimately affects total body homeostasis. A surrogate marker of this homeostasis is acid-base balance, i.e., the concentration of hydrogen ions ([H^+^]) in plasma and other body solutions. Buffering is one of the most important mechanisms of acid-base balance. It corresponds to the less-than-expected response of a system to a specific insult (e.g., upon adding a strong acid to a solution, a buffer reduces the anticipated pH change) [1].

The concept of moderators (buffers) in acid-base physiology was first introduced by Koppel and Spiro in 1914 [2] and further delineated by Van Slyke, who introduced the term less than a decade later [3]. According to these early theories, effective buffers were considered to be mixtures of weak acids (or bases) and their salts. In 1978, Stewart stated that the body’s acid-base status is regulated by three independent variables: strong ion difference (SID), partial pressure of carbon dioxide (PCO_2_) and total non-volatile weak acid concentration ([A_TOT_], mainly albumin) [4]. He further suggested that the buffering capacity can be estimated by the change of the above variables per unit change of [H^+^] or pH [5].

**Figure 1:**
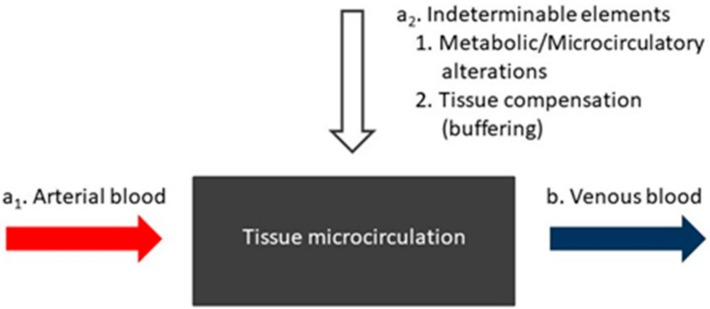
Tissue microcirculation (black box) as a double entry and single exit (arrows) system.

Metabolic derangement and multiple organ dysfunction encountered in sepsis have been, in part, attributed to concomitant impairment of microcirculation [6]. An equation of tissue buffering could be drawn by visualizing tissue microcirculation (black box, Figure 1) as a double-entry (a_1_, a_2_) and single-exit (b) system [1]: a_1_. arterial blood parameters a_2_. indeterminable elements related to tissue injury b. venous blood parameters. 

By comparing the same parameters (of particular importance for the regulation of acid-base balance) at the input and output of a tissue microvascular bed (i.e., arterial and venous blood) and calculating their differences, conclusions could be drawn about: (1) undetectable septic derangements imposed upon the intervening tissues and microcirculation; (2) the body’s capacity to potentially resist such septic interferences with tissue physiological functions and thus limit pH deviations.

There are only limited data concerning the body’s buffering capacity in sepsis. The aim of this study was to assess the buffering capacity in patients with sepsis or septic shock admitted to the intensive care unit (ICU) and to prospectively record probable changes upon clinical improvement or deterioration. Based on Stewart’s view on body fluids interactions [7], the tissue buffering capacity was calculated utilizing the magnitude of SID and/or PCO_2_ change between central venous and arterial blood, corresponding to a specific [H^+^] or pH change.

## 2. Materials and Methods

This is a prospective, observational study, conducted in a 10-bed adult ICU of our institution for 20 months (December 2014 through July 2016). All patients with an admission diagnosis of severe sepsis or septic shock were eligible for entering the study. Seventeen non-septic patients admitted to the same ICU during the study period were used as the controls. A central venous catheter in a subclavian or jugular vein was required by all subjects entering the study in order to obtain blood samples from the superior vena cava. The decision for a central venous line placement and its location (sublclavian, jugular or femoral vein) was made by each patient’s attending physician. Therefore, if the attending physician did not consider it necessary to place a central venous line in a subclavian or jugular vein, the patient was excluded from the study. Written informed consent was obtained from the patients’ next of kin and the study was approved by the Institutional Ethics Committee. 

Severe sepsis and septic shock were defined according to accepted definitions [8] when the study was initiated (2014). The 2016 revised definitions for sepsis and septic shock [9] did not affect patient recruitment for the present study.

The demographics and comorbidities were recorded in all participants upon admission to the ICU. The Acute Physiology and Chronic Health Evaluation (APACHE) II score [10] upon admission and also daily Sequential Organ Failure Assessment (SOFA) scores [11] were calculated.

Routine laboratory and acid-base parameters were also recorded upon ICU admission. The same indices were reassessed in all septic patients, either at sepsis remission / clinical improvement (group A) or upon severe deterioration (group B), defined as elsewhere [12]. 

The definition of sepsis remission includes the following: 1. Restoration of hemodynamic stability (systolic blood pressure ≥90 mmHg and mean blood pressure ≥65 mmHg) for a period of at least 24 h, with no need for vasoactive agents and no tissue hypoperfusion indices, either clinical (e.g., delayed capillary refill time) or laboratory (lactic acid levels <2 mEq/L). 2. The markers of systemic inflammatory response syndrome (SIRS) should have returned to normal, in particular the temperature (T, 36 °C ≤ T ≤ 38 °C) and the white blood cell count (WBC, 4000/µL ≤ WBC ≤ 12,000/μL and ≤10% immature forms). Although the existence of SIRS is no longer used to diagnose sepsis, the above criteria were included as indicators of a subsiding SIRS because (among other) they are monitored by the ICU physicians daily as markers of the progress of a septic episode. Further, the heart rate and respiratory rate were not included because many patients admitted to our ICU suffered from cardiac arrhythmias and/or receive chronotropic agents, while most of them had severe underlying respiratory disorders that did not allow normalization of the respiratory function indices. 3. The organic dysfunctions attributed to the septic episode should have been reversed, and the SOFA score reduced ≥2 points compared to the SOFA score on admission. Irrespectively of the above, if a patient was considered cured by the attending ICU physicians and was discharged, the parameter values on the day of ICU exit were recorded.

The definition of sepsis deterioration includes the following: 1. Significant deterioration of the patient’s hemodynamic status, i.e., on the SOFA severity scale, the patient should score ≥3 points in the cardiovascular system assessment, with an increase in lactate levels (increase > 1mEq/L compared to admission value with an absolute lactate concentration of ≥2 mEq/L) or an increase in the need for vasoactive substances (norepinephrine increase >0.1μg/kg/min or addition of other vasoconstrictors, such as adrenaline and vasopressin). The exceptions were cases where hemodynamic deterioration was due to haemorrhagic shock, obstructive shock, or cardiogenic shock after an acute heart attack. 2. The deterioration of organic deficiencies resulting in an increase in SOFA score ≥1 compared to the admission score.

On the day of ICU admission, while patients were at a semi-recumbent position (30° to 45°), blood samples were drawn simultaneously by two physicians: a) arterial blood from an arterial catheter placed in the radial or the femoral artery; b) venous blood from the superior vena cava, through a central venous catheter inserted in either the subclavian or the internal jugular vein. The blood samples were collected in heparinized 2-mL syringes. Particular care was taken for heparin to be expelled completely from the syringe [13]. PCO_2_, partial pressure of oxygen (PO_2_), lactate concentration ([Lactate^−^]) and pH were measured in a blood gas analyzer (RapidLab®1200 Systems, 2009, Siemens Healthcare Diagnostics Inc., Tarrytown, NY, USA) at 37°C. The plasma concentrations of sodium ([Na^+^]), potassium ([K^+^]) and chloride ([Cl^−^]) were also measured in the blood gas analyzer. The blood gas analyzer uses the electric potential generated by an ion-selective electrode (direct potentiometry) and measures the effective concentration (activity) of electrolytes in plasma water, irrespectively of the protein and lipid levels [14,15]. This measurement is advantageous from the chemical point of view, because activity is the driving force in physiological processes affecting pH [14,15]. In addition, the equilibrium states are determined by the activities of reactants rather than their concentrations [16]. Thus, the activities rather than the concentrations might reflect more accurately the influence of charged electrolyte ions in pH regulation. 

The bicarbonate concentration ([HCO_3_^−^]) was calculated by the Henderson-Hasselbalch equation and the base excess (BE) by the Siggaard-Andersen (van Slyke) equation [17]. The measurement of albumin and phosphate in plasma was conducted in separately drawn blood samples with a Dimension® EXL™ 200 Integrated Chemistry System analyzer (2011, Siemens Healthineers, Newark, Delaware, USA). Routine laboratory tests [complete blood count (CBC), coagulation tests, biochemical parameters, C-reactive protein (CRP) and procalcitonin (PCT)] were daily performed. 

### 2.1. Calculated Acid-Base Variables

The acid-base variables were calculated as follows (all concentrations in mEq/L, unless indicated differently) [18,19,20]:

1. Anion Gap corrected for albumin concentration AG = [Na^+^] – ([Cl^−^] + [HCO_3_^−^]) + 2.5 × {4.5 – [albumin (g/dL)]}
2. Apparent SID (SIDa) = [Na^+^] + [K^+^] − [Cl^−^] − [Lactate^−^]
3. Effective SID (SIDe) = 2.46 × 10 ^pH-8^ × PCO_2_ (mmHg) + [albumin (g/L)] × (0.123 × pH−0.631) + [phosphate(mmol/L)] × (0.309 × pH−0.469)
4. Strong ion gap (SIG) = SIDa – SIDe
5. Inorganic SID = [Na^+^] + [K^+^] − [Cl^−^].

### 2.2. Buffering Capacity

The SIDa and the inorganic SID differences were used for calculating the buffering indices. Differences (Δ) of values between central venous and arterial blood were used for estimation of the buffering capacity [5]:

1. [H^+^] buffering capacity against SID = ΔSID/Δ[H^+^]
2. [H^+^] buffering capacity against PCO_2_ = ΔPCO_2_/Δ[H^+^]
3. pH buffering capacity against SID = ΔSID/Δ[pH]
4. pH buffering capacity against PCO_2_ = ΔPCO_2_/Δ[pH]

The lactate concentration, utilized for SIDa calculation, increases during anaerobic metabolism in sepsis, aggravating acidosis. Therefore, the buffering indices were also calculated in relation to the inorganic SID central venous-arterial differences, since the latter might more accurately reflect the targeted mobilization of strong ion-based mechanisms to counterbalance acidotic disorders.

The buffering capacity for each variable was also calculated as the ratio of variable change (%) to [H^+^] and pH percent change (%) [5], e.g., [H^+^] buffering capacity against SID = %ΔSID/%Δ[H^+^]. 

### 2.3. Statistical Analysis

The categorical variables were analyzed with Fisher’s exact test. The continuous variables were analyzed with Kruskal-Wallis test. Since some measurements were correlated (septic patients on admission and improvement or deterioration), this study implemented generalized estimating equations (GEE), an extension of the generalized linear model that accounts for the within-subject correlation. GEE were used to model the association of buffering indices with explanatory variables. In all GEE models, an unstructured correlation structure was used and the quasi likelihood information criterion (QIC) was used for model selection. The logistic and linear regression models were fitted as appropriate. Fatal outcome in the ICU was modelled with Cox proportional hazards regression models fitting buffering indices as independent variables and the corresponding SOFA scores, to adjust for disease severity. The model fit was assessed by checking plots of residuals. The data analysis was performed with SPSS 17.0 (IBM Corporation, NY, 2008). For all analyses, alpha was set at 0.05 (2-sided).

## 3. Results

A total of 187 patients were admitted to the ICU during the course of the study. Of these, 74 were excluded: 6 re-admissions, 37 due to lack of a blood sample from the superior vena cava and 31 due to lack of informed consent. The study included 113 patients (71 male and 42 female, the mean ± SD age 64.8 ± 14.9 years). All initial comparisons of both continuous and categorical values included sex as a factor. Additionally, in model fitting sex was tested as an independent variable in univariable models as well as the adjusting variable in multivariable models, but it never displayed a significant effect. Of the 187 patients admitted to the ICU during this study, 159 were intubated. Among the 113 patients enrolled in the study, only one was not intubated. The mean (± SD) length of stay (LOS) in the ICU was 17.7 ± 15.0 days and the mean (±SD) duration of mechanical ventilation (MV) was 17.2 ± 14.7 days. The mean (±SD) APACHE II score was 22.7±7.6 and the mean (±SD) SOFA score was 8.6 ± 3.3 on ICU admission. Ninety-six patients (85%) presented with sepsis upon ICU admission and seventeen critically ill, non-septic patients (15%) were used as the controls. Of the 113 patients enrolled in the study, 98 were receiving noradrenaline while 7 of them were also on vasopressin at the time of the study entry. Forty-one patients (36.3%) died in the ICU. The clinical and laboratory characteristics of septic patients are shown in Table 1 and a comparison of the acid-base variables among the septic patient groups is presented in Table 2. Table 3 summarizes the baseline clinical and laboratory characteristics differing significantly between septic and non-septic patients. 

### 3.1. Infection–Involved Pathogens

Seventy nine patients presented with pneumonia, eight patients with bloodstream infection (BSI) (primary BSI in two patients; secondary to pneumonia and pyelonephritis in five and one patients, respectively), two patients with lower limb gangrene, two with mediastinitis (due to esophageal perforation and submandibular abscess), one with mitral valve endocarditis, one with liver abscess, one with peritonitis (after gastric perforation), one with surgical wound infection (post-appendectomy) and one with infectious pericarditis. 

The following pathogens were identified: *Acinetobacter baumannii* (*n* = 19), *Klebsiella pneumoniae* (*n* = 11), *Streptococcus pneumoniae* (*n* = 7), Influenza virus A H1N1 (*n* = 6), *Pseudomonas aeruginosa* (*n* = 3), *Staphylococcus aureus* (*n* = 3), Rhinovirus (*n* = 2), Enterovirus (*n* = 2), *Serratia marcescens* (*n* = 1), *Clostridium subterminale* (*n* = 1), *Stenotrophomonas maltophilia* (*n* = 1), *Escherichia coli* (*n* = 1), Coagulase-negative Staphylococcus (*n* = 1), *Streptococcus anginosus* (*n* = 1), *Enterobacter spp.* (*n* = 1), *Aspergillus fumigatus* (*n* = 1), *Aspergillus niger* (*n* = 1), *Mycobacterium tuberculosis* (*n* = 1), *Legionella pneumophila* (*n* = 1), *Streptococcus constellatus* (*n* = 1), *Prevotella spp.* (*n* = 1), *Propionibacterium spp.* (*n* = 1).

### 3.2. Buffering Indices 

The values of the buffering indices in septic and non-septic patients are presented in Table 4. For the whole cohort of patients, in a GEE linear regression model with ΔPCO_2_/ΔpH as a dependent variable, septic patients on admission displayed significant differences in ΔPCO_2_/ΔpH, compared to non-septic patients: beta coefficient = –47.63, 95% confidence interval (−80.09) – (−15.17), *p* = 0.004, adjusted for disease severity by the corresponding SOFA score. Specifically, upon admission (with non-septic patients as reference category), beta coefficients for ΔPCO_2_/ΔpH were: in septic patients who improved clinically (group A) beta coefficient = −33.21, 95% confidence interval (−65.47) – (−0.95), *p* = 0.044; in septic patients who further progressed to clinical deterioration (group B): beta coefficient = −86.03, 95% confidence interval (−153.35) – (−18.72), *p* = 0.012, adjusted by the corresponding SOFA score. 

### 3.3. Buffering Indices and ICU Fatal Outcome

Table 5 illustrates the Cox proportional hazards models for fatal ICU outcomes. The ΔPCO_2_/ΔpH (%) on admission was significantly associated with a 2% reduction of ICU death hazard in septic patients (when the absolute value of the buffering index decreased). At patient reassessment (remission or deterioration), a one-unit increase of ΔPCO_2_/Δ[H^+^] significantly reduced the ICU death hazard by 44% (disease severity adjusted by the SOFA score).

The inorganic SID buffering indices did not differ between all patient groups and in the septic subjects between admission and reassessment (improvement or deterioration). Lastly, there was no association found with the sepsis outcome. 

### 3.4. Miscellaneous Findings

At deterioration (group B patients), more severe acidosis was observed—as indicated by the BE and pH values (Table 2)—and also albumin levels decreased significantly (Table 1).

The central venous-arterial CO_2_ difference (ΔPCO_2_) increased significantly at clinical improvement in group A patients and decreased at deterioration in group B (Table 2). On admission, ΔPCO_2_ was greater in group B compared to group A patients, however, at a borderline level of statistical significance (*p* = 0.08). There was no significant correlation of ΔPCO_2_ with the gross hemodynamic parameters, SOFA score, dose of norepinephrine administered, [Lactate^−^] and survival.

## 4. Discussion

The present study examined tissue or cellular buffering capacity [7] in patients with sepsis and septic shock at two distinct time points: upon admission to the ICU and upon clinical improvement or severe deterioration. The modifying effect of renal function on strong ion concentration [7], which is not subject to short-term regulation, was ruled out by the study design (venous blood collection from the superior vena cava).

### 4.1. PCO_2_ Buffering Indices

In this study, the buffering capacity was significantly different in septic patients compared to non-septic on admission. Furthermore, it was demonstrated for the first time that the buffering capacity was associated with a fatal ICU outcome in septic patients.

The observed changes in PCO_2_ buffer indices might probably reflect changes in the basal metabolic rate at different stages of sepsis. Kreymann et al. [21] have shown that while in the uncomplicated septic state the basal metabolic rate increases, in severe sepsis and septic shock—accompanied by peripheral tissue hypoperfusion and organ dysfunction—the basal metabolic rate decreases. This finding might indicate body failure in supporting the increased metabolic requirements encountered during a systemic response to infection. An increase in basal metabolic rate occurs during recovery.

CO_2_, a product of mitochondrial metabolism, is treated as one of its surrogate markers, even in the mechanically ventilated ICU patients [22]. In severely septic patients, the reduced CO_2_ production portends imminent death [23]. In addition, mitochondrial metabolism buffers the H^+^ produced during glycolysis and hydrolysis of ATP [24], which are accelerated in sepsis [25]. Therefore, lower ΔPCO_2_/Δ[H^+^] at deterioration might imply failure of metabolic buffering i.e., decreased metabolic activity in mitochondria, that cannot utilize the H^+^ produced by glycolysis/hydrolysis of ATP, thus failing to prevent acidosis. 

The increased risk of death in subjects presenting higher ΔPCO_2_/ΔpH (%) values on admission might suggest mitochondrial hyperactivity for pH regulation, due to the reduced efficiency of mitochondrial metabolism in sepsis (increased proton leak, altered proton pump stoichiometry) [25]. The decreased metabolic efficiency early in sepsis might eventually lead to the failure of handling the proton excess at deterioration and worsening acidosis.

In addition to fluctuations in CO_2_ output, rheological factors may influence the ΔPCO_2_/Δ[H^+^] ([ΔpH]) ratio. Circulatory compromise normally results in increased PCO_2_, [H^+^] and pH arteriovenous gradients [26]. Specifically, in septic patients, reduced tissue perfusion due to low cardiac output and/or microcirculatory alterations have been linked to increased ΔPCO_2_ differences [27,28]. The higher ΔPCO_2_ on admission, in the group of patients who deteriorated compared to those who improved, might indicate tissue hypoperfusion [29] occurring early in sepsis. However, at hemodynamic deterioration ΔPCO_2_ decreased, probably due to the reduced CO_2_ production by mitochondrial metabolism, as mentioned previously. The above findings might be explained by microcirculatory disorders accompanying mitochondrial dysfunction [30] that ultimately lead to metabolic and organic failure [6]. 

ΔPCO_2_ is also likely to increase when anaerobic CO_2_ production increases through buffering of non-volatile acids (such as lactate) by HCO_3_^−^. On the contrary, in this study, hemodynamic deterioration was associated with a significant decrease in ΔPCO_2_, despite the increased need for vasoactive drugs and higher lactate levels (findings consistent with tissue hypoperfusion and anaerobiosis).

### 4.2. Comments on the Utilization of the PCO_2_ Buffering Indices Introduced by Stewart

Τhe specific PCO_2_ buffering indices, introduced by Stewart, are related to the expected change of a solution’s acidity when PCO_2_ changes. For instance, a PCO_2_ rise is expected to increase the [H^+^] (reduce pH). The buffering index represents the PCO_2_ change that leads to one unit increase of [H^+^] (or reduction of the pH) in the solution. The greater this necessary change, the higher the buffering capacity. Therefore, the solution becomes more resistant to attempted [H^+^] (pH) alterations. This might appear to make sense and corresponds perfectly to the chemical behavior of the solution under-study; however, this approach in a way ignores certain elements of human physiology. The present study’s results suggest that changes in this marker should be approached differently, to be consistent with the clinical data. Tissue production of CO_2_ (and thus PCO_2_ difference between arterial and venous blood) indicates mitochondrial metabolism, which normally uses (scavenges) the protons produced during ATP cleavage or oxidation of metabolic substrates (reducing equivalents) for the respiratory chain function. Therefore, the ΔPCO_2_/ΔpH buffering index probably corresponds to the activation degree of mitochondrial metabolism for a unit pH change. Thus, a significant increase may indicate low efficiency of mitochondrial metabolism (e.g., from proton leakage) rather than increased buffering capacity. Other researchers have made similar observations about the suitability of Stewart’s physicochemical approach to interpret acid-base disorders [31]. As it is evident from our study, the integrated physiological function of the body must be taken into consideration, in order to have a comprehensive overview of the pathophysiological phenomena observed.

### 4.3. SID Buffering Indices

In the present study, acidosis worsened at deterioration. According to Stewart’s physicochemical approach, SID changes (independent variable) remain the only mechanism by which body fluids can interact with each other and lead to compensatory changes in [H^+^] (pH) and other dependent variables. Neither of the other two independent variables ([A_TOT_] and PCO_2_) could theoretically compensate the microcirculatory acid-base disorders. The first is not subject to short-term changes, while PCO_2_ values are imposed on tissues by the balance of metabolic CO_2_ production, diffusion, blood flow and lung elimination [7]. In conclusion, the body’s response is directed to the mobilization of regulatory mechanisms that increase SID by changing the concentration of strong ions [7,32].

The SID buffering indices did not differ significantly at the various sepsis stages. This finding was rather expected. The H^+^ concentration disequilibrium across cell membranes (proton electrochemical gradient), vital for cellular metabolic functions, is maintained by the operation of regulatory systems involving the exchange of strong ions [33,34]. Thus, an essentially altered relationship between [H^+^] (or pH) and SID—observed in case of ionic channel disorders—might interfere with cell signaling and metabolic pathway regulation. This might represent an exhaustion of compensatory mechanisms in acidotic disorders, leading rapidly to death.

### 4.4. Limitations

This study presents certain limitations, the relatively small number of patients enrolled in the study being the first one. Therefore, it was preferred to focus on the arising differences of means (the shift in location of the compared distributions) in a distribution-free way, rather than pursuing comparisons of precise estimates, which would not yield robust results. Studies with larger samples would allow inferences based on distributional estimates. Surgical patients were underrepresented in our patient-population sample due to the nature of our institution (primarily Internal Medicine departments). Lastly, tissue buffering was assessed at the upper part of the body, as no blood specimens were drawn from the lower part of the body and the entire visceral circulation, thus bypassing the influence of renal function.

Further studies are needed for external validation of our findings in different case-mixes. Moreover, it would be interesting to assess the buffering capacity of the body overall, as well as separately, per organ and system.

## 5. Conclusions

In the particular cohort of patients studied, a difference in buffering capacity was recorded between septic and non-septic patients on admission. The PCO_2_ buffering indices were significantly associated with fatal ICU outcome.

## Figures and Tables

**Table 1:** Clinical and laboratory characteristics of septic patients at ICU admission and upon clinical improvement or deterioration (*n* = 79) ^*,†.^

Variable	Group A (*n* = 52)			Group B (*n* = 27)		
	Admission	Sepsis Remission	*p* Value	Admission	Sepsis Deterioration	*p* Value
APACHE II score	21.5 ± 7.8^b^	NA	NA	26.7 ± 7.1^b^	NA	NA
SOFA score	8.5 ± 3.1	2.2 ± 2.4	<0.001	10.4 ± 3.0	13.4 ± 3.0	<0.001
SaO_2_ (%)	96.5 ± 2.7	96.5 ± 1.8	0.42	95.8 ± 2.8	91.5 ± 7.3	<0.001
SvO_2_ (%)	76.0 ± 7.4	65.4 ± 10.6	<0.001	74.7 ± 9.8	69.3 ± 7.7	0.02
PO_2_/FiO_2_	203 ± 94	279 ± 99	<0.001	181 ± 92	158 ± 108	0.2
Hemoglobin (g/dL)	11.4 ± 2.0	9.2 ± 1.5	<0.001	11.1 ± 1.8	9.6 ± 1.8	0.001
WBC (×10^3^/μL)	16.55 ± 9.3	10.8 ± 4.1	<0.001	20.1 ± 13.4	21.5 ± 12.7	0.23
PMN (%)	84 ± 13	76 ± 8	<0.001	84 ± 9	86 ± 9	0.18
PLT (×10^3^/μL)	238.2 ± 92.4	316.7 ± 158.5	0.009	248.7 ± 119.0	138.8 ± 100.7	0.002
Fibrinogen (mg/dL)	588 ± 189	582 ± 159	0.34	608 ± 220	523 ± 226	0.29
aPTT (sec)	44.0 ± 14.6	44.3 ± 10.5	0.43	43.9 ± 8.8	59.5 ± 28.1	0.004
INR	1.35 ± 0.32	1.46 ± 1.68	0.64	1.60 ± 0.80	2.12 ± 1.59	0.09
D-Dimers (μg/mL)	4.15 ± 3.47	3.33 ± 2.37	0.08	8.73 ± 8.74	6.87 ± 3.61	0.92
Urea (mg/dL)	68.4 ± 43.7	63.4 ± 33.1	0.61	100.6 ± 67.1	86.4 ± 38.3	0.43
Creatinine (mg/dL)	1.36 ± 1.19	0.89 ± 0.60	<0.001	1.76 ± 1.32	1.49 ± 0.81	0.43
Bilirubin (mg/dL)	0.86 ± 1.21	0.79 ± 1.56	0.002	1.07 ± 1.14	1.36 ± 1.26	0.08
Albumin (g/dL)	2.26 ± 0.52	2.20 ± 0.49	0.6	2.16 ± 0.45	1.71 ± 0.52	0.001
CRP (mg/dL)	29.3 ± 49.1	9.9 ± 16.9	0.01	20.4 ± 16.6	17.7 ± 14.5	0.75
Procalcitonin (ng/mL)	5.75 ± 6.73	0.57 ± 0.64	<0.001	2.88 ± 3.64	1.53 ± 1.00	0.47
Norepinephrine (μg/kg/min)	0.143 ± 0.106	0.004 ± 0.001	<0.001	0.258 ± 0.166	0.532 ± 0.315	<0.001

* Values are mean ± SD; ^†^ *p* <.001 (means compared at admission) APACHE: Acute Physiology And Chronic Health Evaluation; SOFA: Sequential Organ Failure Assessment; SaO_2_: hemoglobin oxygen saturation in arterial blood; SvO_2_: hemoglobin oxygen saturation in central venous blood; PO_2_/FiO_2_: arterial partial oxygen pressure to fractional inspired oxygen ratio; WBC: white blood cell count; PMN: polymorphonuclear cells; PLT: platelets; aPTT: activated partial thromboplastin time; INR: international normalized ratio; CRP: C-reactive protein; NA: non-applicable.

**Table 2:** Comparisons of acid-base variables in septic patients (*n* = 79) ^*,†.^

Variable	Group A (*n* = 52)			Group B (*n* = 27)		
	Admission	Sepsis Remission	*p* Value	Admission	Sepsis Deterioration	*p* Value
pH_a_	7.37 ± 0.073	7.47 ± 0.05	<0.001	7.35 ± 0.09	7.23 ± 0.16	0.001
pH_v_	7.34 ± 0.07	7.42 ± 0.05	<0.001	7.32 ± 0.08	7.20 ± 0.15	<0.001
PCO_2a_ (mmHg)	45.3 ± 10.3	39.1 ± 9.7	0.001	43.4 ± 7.7	45.1 ± 12.4	0.61
PCO_2v_ (mmHg)	51.8 ± 10.3	46.5 ± 10.1	0.010	51.1 ± 7.6	51.4 ± 11.0	0.91
[HCO_3_^–^_a_] (mEq/L)	25.4 ± 4.9	27.3 ± 5.1	0.02	23.6 ± 5.4	19.5 ± 6.8	0.03
[HCO_3_^–^_v_] (mEq/L)	27.0 ± 4.8	29.3 ± 4.9	0.005	25.9 ± 5.8	20.7 ± 6.7	0.008
[Lac_a_] (mEq/L)	1.3 ± 0.5	1.1 ± 0.4	0.02	2.4 ± 1.3	5.2 ± 3.8	0.001
[Lac_v_] (mEq/L)	1.3 ± 0.6	1.2 ± 0.4	0.1	2.5 ± 1.3	4.9 ± 3.4	0.002
AG_a_ (mEq/L)	13.27 ± 2.94	12.58 ± 3.34	0.07	14.95 ± 3.19	15.38 ± 6.36	0.61
AG_v_ (mEq/L)	13.39 ± 2.95	12.55 ± 3.04	0.07	14.55 ± 3.22	15.62 ± 6.29	0.58
BE_a_ (mEq/L)	–0.29 ± 4.75	3.20 ± 4.48	0.001	–1.79 ± 6.24	–8.04 ± 8.19	0.004
BE_v_ (mEq/L)	0.68 ± 4.62	4.11 ± 4.33	<0.001	–0.41 ± 6.50	–6.85 ± 9.14	0.006
[Cl_a_] (mEq/L)	106.4 ± 5.7	105.7 ± 7.1	0.39	105. 1 ± 6.5	109.0 ± 4.0	0.005
[Cl_v_] (mEq/L)	105.0 ± 5.7	104.0 ± 6.9	0.23	103.4 ± 6.9	107.8 ± 4.4	0.004
[Na_a_] (mEq/L)	139.5 ± 5.4	139.8 ± 5.0	0.78	137.8 ± 4.8	136.9 ± 3.8	0.54
[Na_v_] (mEq/L)	139.9 ± 5.4	140.1 ± 4.7	0.84	138.0 ± 5.1	137.1 ± 4.7	0.61
SID_a_ (mEq/L)	35.7 ± 4.6	36.8 ± 5.3	0.15	34.7 ± 5.5	27.4 ± 6.4	<0.001
SID_v_ (mEq/L)	37.4 ± 4.5	38.8 ± 5.2	0.06	36.6 ± 6.1	29.1 ± 6.6	0.001
SIDe_a_ (mEq/L)	34.0 ± 4.7	35.6 ± 5.4	0.06	32.7 ± 5.6	27.5 ± 6.6	0.009
SIDe_v_ (mEq/L)	35.8 ± 4.5	37.6 ± 5.3	0.03	35.1 ± 6.1	28.7 ± 6.5	0.002
SIG_a_ (mEq/L)	1.69 ± 3.17	1.24 ± 3.17	0.3	1.98 ± 2.59	–0.15 ± 3.40	0.03
SIG_v_ (mEq/L)	1.62 ± 2.67	1.22 ± 3.08	0.3	1.49 ± 2.73	0.34 ± 3.60	0.18
ΔPCO_2_v-a (mmHg)	6.47 ± 3.03	7.41 ± 3.24	0.05	7.71 ± 3.07	6.30 ± 3.23	0.04

* Values are mean ± SD; ^†^ (_a_) and (_v_) suffixes indicate measurements in arterial or central venous blood, respectively. PCO_2_: partial pressure of carbon dioxide; [HCO_3_^-^]: bicarbonate concentration; [Lac]: lactate concentration; AG: anion gap corrected for albumin; BE: base excess; [Cl]: chloride concentration; [Na]: sodium concentration; SID: apparent strong ion difference; SIDe: effective strong ion difference; SIG: strong ion gap; ΔPCO_2_v-a: central venous-arterial difference of carbon dioxide partial pressure.

**Table 3:** Comparisons of clinical and laboratory characteristics in non-septic and septic patients (*n* = 113) ^*.^

Variable	Non Septic (*n* = 17)	Septic (*n* = 96)	*p* Value
Age (years)	62.5 ± 13.4	65.2 ± 15.2	0.37
APACHE score	18.5 ± 5.8	23.5 ± 7.7	0.01
SOFA score	6.0 ± 2.9	9.1 ± 3.2	<0.001
PO_2_/FiO_2_	317 ± 102	190 ± 90	<0.001
Norepinephrine (μg/kg/min)	0.076 ± 0.162	0.198 ± 0.209	<0.001
Hemoglobin (g/dL)	10.2 ± 1.9	11.3 ± 2.0	0.04
WBC (× 10^3^/μL)	11.6 ± 4.0	17.9 ± 10.5	0.008
Fibrinogen (mg/dL)	450 ± 180	589 ± 195	0.01
Albumin (g/dL)	2.6 ± 0.5	2.2 ± 0.5	0.01
CRP (mg/dL)	8.1 ± 8.5	25.4 ± 39.4	0.002
Procalcitonin (ng/mL)	0.93 ± 1.36	5.65 ± 7.92	0.01
pH	7.44 ± 0.07	7.36 ± 0.09	<0.001
BE (mEq/L)	0.35 ± 5.43	–0.95 ± 5.31	0.41
[Lac] (mEq/L)	1.4 ± 1.4	1.7 ± 1.1	0.02
AG (mEq/L)	13.18 ± 3.13	13.94 ± 3.44	0.54
SID (mEq/L)	34.9 ± 5.7	35.6 ± 5.0	0.50
SIG (mEq/L)	1.10 ± 2.51	1.96 ± 3.35	0.47
ΔPCO_2_ (mmHg)	6.9 ± 2.9	6.7 ± 3.5	0.99

* Values are mean ± SD. APACHE: acute physiology and chronic health evaluation; SOFA: sequential organ failure assessment; PO_2_/FiO_2_: partial pressure of oxygen to fractional oxygen concentration ratio; WBC: white blood cell count; CRP: C-reactive protein; BE: base excess; [Lac]: lactate concentration; AG: anion gap corrected for albumin; SID: apparent strong ion difference in arterial blood; SIG: strong ion gap in arterial blood; ΔPCO_2_: central venous-arterial difference of carbon dioxide partial pressure.

**Table 4:** Buffering indices in septic and non-septic patients (*n* = 96) ^*,†^.

Variables	Non Septic (*n*=17)	Group A (*n*=52)		Group B (*n*=27)	
		Admission	Sepsis remission	Admission	Sepsis deterioration
ΔSID/ΔpH, (mEq/L/unit pH)	−56.7 ± 30.4	−63.2 ± 62.9	−56.0 ± 64.5	−53.5 ± 65.1	−63.1± 96.9
ΔSID/ΔpH (%)	−12.3 ± 6.5	−13.5 ± 12.7	−11.6 ± 12.7	−11.0 ± 14.6	−18.2 ± 26.2
ΔSID/Δ[H^+^], (mEq/nmol/L)	0.66 ± 0.34	0.58 ± 0.53	0.68 ± 0.79	0.53 ± 0.58	0.46 ± 0.69
ΔSID/Δ[H^+^] (%)	0.69 ± 0.37	0.76 ± 0.72	0.65 ± 0.73	0.63 ± 0.86	1.07 ± 1.56
ΔPCO_2_/ΔpH, (mmHg/unit pH)	−183.3 ± 34.5	−224.9 ± 102.7	−192.4 ± 96.0	−283.7 ± 178.8	−198.6 ± 128.1
ΔPCO_2_/ΔpH (%)	−38.1 ± 7.5	−36.8 ± 14.2	−37.8 ± 18.3	−48.3 ± 27.7	−35.0 ± 23.4
ΔPCO_2_/Δ[H^+^], (mmHg/nmol/L)	2.08 ± 0.31	2.12 ± 0.79	2.32 ± 1.18	2.65 ± 1.55	1.44 ± 0.87
ΔPCO_2_/Δ[H^+^] (%)	2.13 ± 0.44	2.05 ± 0.79	2.10 ± 1.06	2.76 ± 1.64	2.03 ± 1.42

In a GEE linear regression model with ΔPCO_2_/Δ[H^+^] as dependent variable, group B septic patients displayed significant difference in ΔPCO_2_/Δ[H^+^] on clinical deterioration, compared to non-septic patients: beta coefficient = – 0.58, 95% confidence interval (–0.94) – (–0.23), *p* = 0.001, adjusted by the corresponding SOFA score.

**Table 5:** Cox proportional hazards regression for fatal ICU outcome (*n* = 96).

Univariable Models	Hazard Ratio	95% Confidence Interval	*p* Value
Admission ΔPCO_2_/ΔpH (%)	0.98	0.97–0.99	0.01
Admission ΔPCO_2_/Δ[H^+^] (%)	1.39	1.09–1.77	0.008
Remission/deterioration ΔSID/ΔpH (%)	0.98	0.96–0.99	0.03
Remission/deterioration ΔSID/Δ[H^+^] (%)	1.46	1.07–2.00	0.02
Remission/deterioration ΔPCO_2_/Δ[H^+^] (mmHg/nmol/L)	0.41	0.27–0.64	<0.001
Models adjusted for SOFA score *	Hazard ratio	95% confidence Interval	*p* value
Admission ΔPCO_2_/ΔpH (%)	0.98	0.97–0.99	0.02
Admission ΔPCO_2_/Δ[H^+^] (%)	1.27	0.98–1.65	0.07
Remission/deterioration ΔPCO_2_/Δ[H^+^] (mmHg/nmol/L)	0.56	0.33–0.96	0.03

* SOFA score at all time points (admission, sepsis remission/ deterioration) was an independent predictor of death in the ICU with hazard ratios ranging between 1.21 and 1.43 with high statistical significance (*p* values ranging between <0.001 and 0.003). Δ corresponds to the difference of the related variable between central venous and arterial blood; PCO_2_: partial pressure of carbon dioxide; [H^+^]: hydrogen cation concentration; SID: apparent strong ion difference; SOFA: sequential organ failure assessment.
